# Can interoceptive attentiveness modulate the brain correlates of observation of pain in others? A fnirs study

**DOI:** 10.1192/j.eurpsy.2021.1164

**Published:** 2021-08-13

**Authors:** L. Angioletti, M. Balconi

**Affiliations:** International Research Center For Applied Cognitive Neuroscience (irccan), Research Unit In Affective And Social Neuroscience, Department Of Psychology, Catholic University of the Sacred Heart, Milan, Italy, Milano, Italy

**Keywords:** interoceptive attentiveness, Pain, empathy, fNIRS

## Abstract

**Introduction:**

Empathizing with others’ pain appears to recruit the whole pain matrix, including a collection of frontal regions involved in the affective, motivational, cognitive, and attentional dimension of pain.

**Objectives:**

This research explored how the modulation of interoceptive attentiveness (IA) can influence the frontal (dorsolateral prefrontal cortex -DLPFC- and somatosensory cortices) activity related to the emotional regulation and sensory response of observing pain in others.

**Methods:**

22 healthy participants were required to observe face versus hand, painful/non-painful stimuli in an individual versus social condition while brain hemodynamic response (oxygenated [O2Hb] and deoxygenated hemoglobin [HHb] components) was measured by functional Near-Infrared Spectroscopy (fNIRS). The sample was divided into experimental (EXP) and control (CNT) groups and the EXP group was explicitly required to focus on its interoceptive correlates while observing the stimuli.

**Results:**

In the individual condition, higher brain responsiveness was detected for painful confronted to non-painful stimuli, and a left/right hemispheric lateralization was found for the individual and social condition, respectively. Besides, both groups showed higher DLPFC activation for face stimuli displayed in the individual condition compared to hand stimuli in the social condition. However, face stimuli activation prevailed for the EXP group, suggesting the direct interoceptive phenomenon has certain features, namely it manifests itself in the individual condition and for pain stimuli.

**Conclusions:**

We can conclude that IA modulation promoted the recruitment of internal adaptive regulatory strategies engaging both DLPFC and somatosensory regions towards emotionally relevant stimuli (painful faces displayed in the individual condition). Therefore IA could be trained for promoting emotion regulation and empathic response.

